# Peroxide-Mediated Oxygenation of Organic Compounds by Fungal Peroxygenases

**DOI:** 10.3390/antiox11010163

**Published:** 2022-01-14

**Authors:** Martin Hofrichter, Harald Kellner, Robert Herzog, Alexander Karich, Jan Kiebist, Katrin Scheibner, René Ullrich

**Affiliations:** 1 Department of Bio- and Environmental Sciences, TU Dresden-International Institute Zittau, Markt 23, 02763 Zittau, Germany; harald.kellner@tu-dresden.de (H.K.); robert.herzog@tu-dresden.de (R.H.); alexander.karich@tu-dresden.de (A.K.); rene.ullrich@tu-dresden.de (R.U.); 2 Institute of Biotechnology, Brandenburg University of Technology Cottbus-Senftenberg, Universitätsplatz 1, 01968 Senftenberg, Germany; jan.kiebist@izi-bb.fraunhofer.de (J.K.); katrin.scheibner@b-tu.de (K.S.); 3 Fraunhofer Institute for Cell Therapy and Immunology, Branch Bioanalytics and Bioprocesses, Am Mühlenberg 13, 14476 Potsdam-Golm, Germany

**Keywords:** unspecific peroxygenases, UPO, EC 1.11.2.1, monooxygenases, peroxidases, hydroxylation, epoxidation, dealkylation

## Abstract

Unspecific peroxygenases (UPOs), whose sequences can be found in the genomes of thousands of filamentous fungi, many yeasts and certain fungus-like protists, are fascinating biocatalysts that transfer peroxide-borne oxygen (from H_2_O_2_ or R-OOH) with high efficiency to a wide range of organic substrates, including less or unactivated carbons and heteroatoms. A twice-proline-flanked cysteine (PCP motif) typically ligates the heme that forms the heart of the active site of UPOs and enables various types of relevant oxygenation reactions (hydroxylation, epoxidation, subsequent dealkylations, deacylation, or aromatization) together with less specific one-electron oxidations (e.g., phenoxy radical formation). In consequence, the substrate portfolio of a UPO enzyme always combines prototypical monooxygenase and peroxidase activities. Here, we briefly review nearly 20 years of peroxygenase research, considering basic mechanistic, molecular, phylogenetic, and biotechnological aspects.

## 1. Introduction

According to enzyme nomenclature, unspecific peroxygenases (UPOs, EC 1.11.2.1) belong to the sub-subclass of oxidoreductases that act ‘with H_2_O_2_ as acceptor, one oxygen atom of which is incorporated into the product’ (https://www.qmul.ac.uk/sbcs/iubmb/enzyme/EC1/11/2; (accessed on 20 December 2021) [1]). They bear a cysteine-ligated heme in the active site (heme-thiolate proteins/HTP) and behave ‘promiscuously’ with respect to oxygen transfer reactions, i.e., one individual UPO protein may catalyze dozens of different oxyfunctionalizations in dependence on the geometry of its heme access channel and the structure of the particular substrate molecules [2,3,4]. The first UPO was discovered as an ‘untypical’ veratryl-alcohol-oxidizing haloperoxidase in the Black Poplar mushroom (*Agrocybe aegerita*, nowadays *Cyclocybe aegerita*) [5], a hardwood and leaf-litter-dwelling edible fungus belonging to the large basidiomycetous order of Agaricales (which typically form mushrooms and ‘toadstools’ as fruiting organs) [6]. More wild-type UPOs were later found in cultures of other filamentous fungi (including the ink-cap *Coprinellus radians*, the Pinwheel mushroom *Marasmius rotula*, and the ubiquitous ascomycetous mold *Chaetomium globosum*) [7,8]. Moreover, chloroperoxidase (CPO, EC 1.11.1.10), secreted by the ascomycetous sooty mold *Caldariomyces fumago*, which had been a ‘phylogenetic and catalytic orphan’ for a long time, turned out to be a special type of UPO [8,9,10].

Though UPOs oxygenate diverse substrates simply with peroxide as a co-substrate, the catalyzed oxyfunctionalizations proceed mostly with high selectivity. In addition, the UPOs tested so far have shown remarkable insensitivity regarding organic solvents and towards several physicochemical factors, making them interesting catalysts for biotechnology and organic synthesis. The major drawback remains their limited availability (there are no commercial UPOs on the market) and there are only 20 UPOs available even at the laboratory scale (see Table 1 below). A second shortcoming is their sensitivity towards high peroxide concentrations, which causes enzyme inactivation. While the former problem may be solved in the near future by new and optimized expression techniques, the second issue is subject to intelligent reaction design and improved peroxide supply under reaction conditions.

Key findings reported here are the outcome of five integrated projects of the European Union (BIORENEW, PEROXICATS, INDOX, ENZOX2, SUSBIND) as well as of several German and Spanish national projects with participants from academia, research institutions and industry of nine European countries as well as the US [11,12,13,14]. This includes basic information on the structure, function and phylogeny of UPOs, as well as approaches addressing their biotechnological potential. Respective findings have been published in around 100 scientific articles and dozens of patents since 2004 [5,15,16,17,18,19,20,21,22,23]; previous reviews documenting the then-current state of peroxygenase research or addressing specific aspects of peroxygenase catalysis and expression were published in 2006, 2010, 2014, 2015, 2019, 2020, and 2021 [2,8,10,24,25,26,27,28,29,30,31].

## 2. UPO Production and Purification

The first UPO of *Cyclocybe* (syn. *Agrocybe*) *aegerita* was found while cultivating the fungus in an agitated soybean-based complex medium [5]. Three major isoforms of this secreted enzyme, oxidizing various aryl alcohols, phenolics, ABTS, and halides, were present in the culture broth and could be separated and purified to homogeneity by several ultrafiltration and chromatographic steps using ion exchangers and FPLC devices [32]. Meanwhile, the genome of *C*. *aegerita* has been sequenced, revealing 18 different UPO genes [33]. Further screenings in complex media with varying amounts of soybean flour and soy extracts, yeast extract, peptones of different origin, alfalfa pellets, and/or sugars (glucose, fructose) led to the discovery of more UPOs from agaric Basidiomycota (wood and leaf-litter dwellers), e.g., *Cyclocybe* (*Agrocybe*) *parasitica* (unpublished result), *Marasmius rotula* [34] and *M*. *wettsteinii* [35], *Coprinellus radians* and *Coprinopsis verticillata* [36], and *Candolleomyces* (*Psathyrella*) *aberdarensis* [29,37]. The production of an ascomycetous wild-type UPO in appreciable amounts has so far only been successful in the case of the cellulolytic mold *Chaetomium globosum* [38]. Molecular masses and isoelectric points of characterized wild-type UPOs range from 23 to 64 kDa and from pH 4.0 to 9.0, respectively (Table 1). As far as studied, *N*-glycosylation at different sites of the apo-protein is of the ‘high-mannose’ type and can account for up to 20% of the mass of the mature enzyme [39,40].

**Figure 1:**
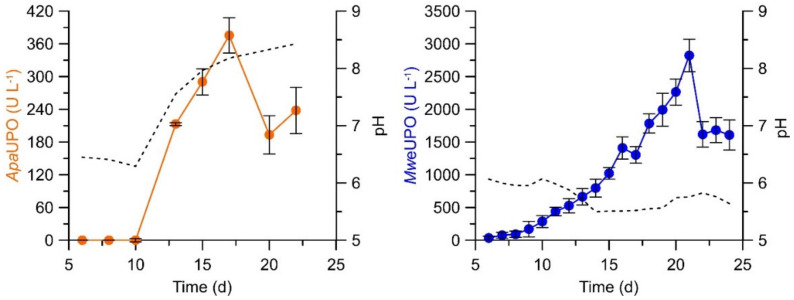
UPO activities of *Cyclocybe* (*Agrocybe*) *parasitica* (*Apa*UPO) and *Marasmius wettsteinii* (*Mwe*UPO) measured with veratryl alcohol (which is oxidized into veratraldehyde) in agitated liquid cultures (500 mL flasks, 100 rpm, medium: 20 g L^−1^ soybean flour suspended in water).

The UPO concentrations secreted by these wild-type fungi in surface or agitated batches can greatly vary, not only in dependence on the particular fungal strain and culture conditions but also from cultivation approach to cultivation approach (between 2 and 600 mg L^−1^, corresponding to 50–40,000 U L^−1^ veratryl alcohol oxidation units) [5,34,36,38,41]. The latter suggests that—in addition to external culture conditions (i.e., composition and agitation of the liquid medium, dioxygen supply, pH, temperature, etc.)—a kind of autoregulation (‘internal clock’) in the fungus may influence UPO synthesis and secretion. As an example, Figure 1 describes the time courses of UPO activity in cultures of *Cyclocybe (Agrocybe*) *parasitica* and *Marasmius wettsteinii* (unpublished results).

All studies carried out to date indicate that fungal wild-type strains secrete ‘short’ UPOs of family I in higher amounts than ‘long’ UPOs of family II (with regard to UPO families, compare the phylogeny below) (Table 1). Efforts to improve homologous UPO production are still ongoing, particularly with respect to the identification of specific elicitor/inducer molecules and the use of naturally fast-growing Ascomycota (molds) and basidiomycetous yeasts with UPO genes in their genomes (e.g., *Aspergillus*, *Chaetomium*, *Trichoderma*, *Rhodotorula*). Although several attempts have been made to identify specific substances and conditions that increase UPO levels, it is still unclear which mechanisms actually trigger their production and secretion [42]. More importantly, it should be noted that not only is the total amount of UPO protein (mg) formed in a batch important, but also its specific activity (U mg^−1^) as a relative proportion (% of total protein) in the original culture liquid, since this is a measure for the natural UPO ‘enrichment’ during cultivation. In particular, in culture media that are rich in peptides and proteins (soybean components, peptones), these values can be unfavorable compared to synthetic media.

In addition to homologously produced wild-type UPOs, a steadily increasing number of recombinant UPOs have become available over the last few years. Thus far, they belong to 14 fungal species and the expression hosts are molds (*Aspergillus oryzae*, *A. nidulans*, *A. niger*), ascomycetous yeasts (*Saccharomyces cerevisiae* and methylotrophic *Pichia* (*Komagataella*) *pastoris*), as well as the Gram-negative bacterium *Escherichia coli* [8] (Table 1). The world’s leading enzyme supplier, Novozymes A/S (Copenhagen, DK), prepared the first recombinant UPO from the ink-cap *Coprinopsis cinerea* in *A. oryzae* and tested its suitability for oxyfunctionalizing syntheses [12,20]; later, a second recombinant UPO from the thermophilic soil ascomycete *Humicola insolens* (syn. *Mycothermus thermophilus*, *Scytalidium thermophilum*) was produced using the same host [38,43,44].

Miguel Alcalde and co-workers were the first who successfully applied directed evolution in the yeast *S. cerevisiae* to generate UPO mutants with improved synthetic properties, among them Pada-I, Jawa, and Solo, *Aae*UPO mutants with better performance and decreased peroxidative activity. The Pada-I variant can be produced at levels of up to 300 mg L^−1^ in a second yeast, *Pichia pastoris* (tandem-expression system), and is useful when the targeted products (or already the substrates) bear reactive phenolic groups that would rapidly undergo oxidation by the peroxidative activity of UPO (one-electron oxidation) [45,46,47,48]. The latter leads to the formation of phenoxy radicals and subsequently to undesired coupling products and quinones. This shortcoming can be circumvented by applying radical scavengers such as ascorbic acid, which reduce the radicals formed [49,50], but engineered UPOs offer a more elegant way to prevent these side activities [51]; furthermore, these molecular techniques can be applied to improve other UPO shortcomings, such as stability in organic solvents [52].

**Table 1:** Survey on wild-type and recombinant UPOs from 18 fungal species available so far.

Enzyme	Donating Organism	Expression System	Mutant	Molecular Weight (kDa)	Secretion Level (mg L^−1^)	Ref.
*Aae*UPO	*Agrocybe aegerita* (syn. *Cyclocybe*)	Wild-type	No	45–46 (37 *)	10	[5,32,39]
r*Aae*UPO (PaDa-I)	*A. aegerita*	*Saccharomyces cerevisiae*, *Pichia pastoris*	Yes	51	300	[45,46,53]
r*Aae*UPO	*A. aegerita*	*P. pastoris*	No	-	290	[54]
r*Ani*UPO	*Aspergillus niger*	*P. pastoris*	No	60	-	[55]
r*Cci*UPO	*Coprinopsis cinerea*	*Aspergillus oryzae*	No	44	-	[12,18]
*Cgl*UPO	*Chaetomium globosum*	Wild-type	No	36	44	[38]
r*Cgl*UPO	*C. globosum*	*A. oryzae*	No	-	-	[18]
CPO (*Lfu*UPO)	*Leptoxyphium fumago* (syn. *Caldariomyces*)	Wild-type	No	40–42	600	[56,57,58]
r*Lfu*UPO	*L. fumago*	*A. niger*	No	45-50	10	[59]
*Cra*UPO	*Coprinellus radians*	Wild-type	No	43–45 (27 *)	-	[36]
*Cve*UPO	*Coprinopsis verticillata*	Wild-type	No	40 (23 *)	-	[41]
r*Cvi*UPO	*Collariella virescens*	*A. oryzae*, *Escherichia coli*	No	-	7	[18,60]
r*Dca*UPO	*Daldinia caldariorum*	*A. oryzae*, *E. coli*	No	-	3	[18,60]
r*Hin*UPO	*Humicola insolens*	*A. oryzae*	No	-	-	[18,38]
*Hsp*UPO	*Hypoxylon sp.* EC38	*S. cerevisiae*	No	55 (28 *)	200	[61]
*Mro*UPO	*Marasmius rotula*	Wild-type	No	32 (27 *); 64 **	450	[34,62]
*Mwe*UPO	*M. wettsteinii*	Wild-type	No	32; 62 **	-	[35]
r*Mfe*UPO	*Myceliophthora fergusii*	*A. oryzae*	No	-	-	[18]
r*Mhi*UPO	*M. hinnulea*	*A. oryzae*	No	-	-	[18]
*Pab*UPO	*Candolleomyces aberdarensis (*syn. *Psathyrella aberdarensis)*	Wild-type	No	40–41	-	[29,63]
r*Pab*UPO-I (Grogu)	*C. aberdarensis*	*S. cerevisiae* *P. pastoris*	Yes Yes	47 -	14 290	[64] [64]
r*Pab*UPO-II	*C. aberdarensis*	*S. cerevisiae*	No	45	5.4	[64]
r*Pvi*UPO	*Pestalotiopsis virgatula*	*A. oryzae*	No	-	-	[18]
r*Thy*UPO	*Thielavia hyrcaniae*	*A. oryzae*	No	-	-	[18]

* Deglycosylated protein; ** dimer.

Using the *Saccharomyces*-*Pichia* expression system, UPOs from *A. aegerita*, *Marasmius rotula* (M. Alcalde, personal communication), and *Candolleomyces aberdarensis* (r*Pab*UPO with interesting pH stability) have been produced at levels of up to 300 mg L^−1^ [54,64].

A modified *Saccharomyces*-*Pichia* system has recently been applied to express four active peroxygenases (r*Mro*UPO, r*Cgl*UPO, r*Mth*UPO, and r*Tte*UPO; Table 1) using a ‘Golden Gate platform’ consisting of three modules (signal peptide library—module 1, UPO genes—module 2, and protein tags—module 3) [65,66]. The same group used the *Saccharomyces* system to engineer one of the newly expressed enzymes (r*Mth*UPO) from the sordariomycete *Myceliophthora thermophila* (*Corynascus heterothallicus*) [67]. Notably, this fungus has been a source of industrial enzymes (e.g., cellulase, pectinases, laccase) for many years [68,69].

All in all, for the time being, the *Pichia* expression system [70] seems to be the production system of choice, and it has been demonstrated to be applicable also without pre-engineering in *Saccharomyces*, e.g., for a UPO of the soft-rot fungus *Hypoxylon* sp. EC38 [61], as well as for the aforementioned UPOs from *C. aberdarensis* and a UPO from an *Aspergillus niger* strain [55,64]. Pilot-scale production (2500 L) of recombinant *Aae*UPO with *Pichia* has recently been demonstrated, resulting in over 700 g enzyme within six days [54].

Angel T. Martínez and co-workers have developed an expression system based on *E. coli* as a bacterial host. They were able to express native and mutated UPOs from *Collariella virescens* (syn., *Chaetomium virescens*) and *Daldinia caldariorum* in this system, which have not been available as wild-type enzymes before [22,60]. Recombinant UPOs produced in *E. coli*, however, are still limited in the obtained amounts and lacking glycosylation, which may affect physicochemical properties such as water solubility and pH stability. On the other hand, UPO properties may also change when they are expressed in *S. cerevisieae* or *P. pastoris*. For example, the molecular mass of recombinant *Aae*UPO (Pada-I) increased from 45–46 kDa to 51–52 kDa due to over-glycosylation (Table 1). Such different or lacking glycosylation can even affect the kinetic parameters, as has been already observed for fungal aryl alcohol oxidases [71].

A valuable summary on the heterologous expression of unspecific peroxygenases in all available hosts as well as in wild-type fungi has recently been published by [72]. Despite all undoubted efforts in the field of heterologous expression, there is no commercial UPO preparation available at present. It is conceivable, however, that this bottleneck will be overcome in the near future by further improving existing expression systems and by developing new expression strategies, e.g., cell-free approaches based on isolated ribosomes [23,73,74].

## 3. UPO Characteristics and Catalytic Cycles

**Figure 2:**
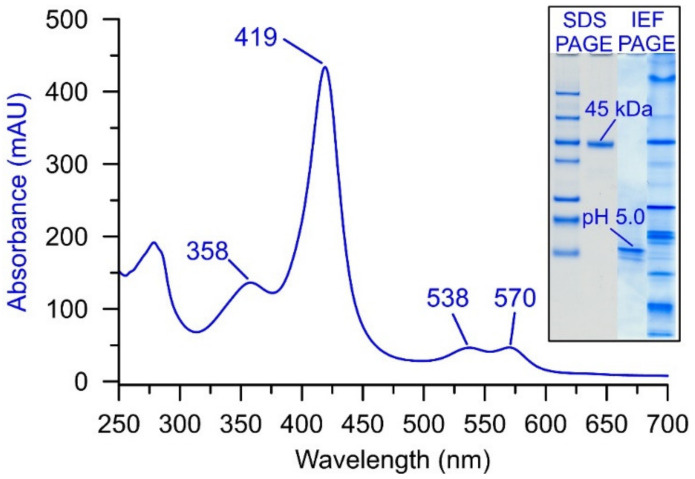
UV–Vis spectrum of a purified wild-type UPO from *Cyclocybe* (*Agrocybe*) *parasitica* (*Apa*UPO) with the characteristic Soret band at 419 nm and the α, β, and δ bands at 570, 538, and 358 nm, respectively. The inset shows the corresponding electrophoresis results: SDS-PAGE (left lanes) with molecular mass standards and the 45 kDa band; isoelectric focusing (IEF)-PAGE (right lanes) with the indicative pI band at pH 5.0 and IEF markers.

All UPOs purified and characterized so far have characteristic absorption properties in the visible and UV range, with a striking maximum (Soret band) in the ground state (native enzyme) between 416 and 421 nm. Additional local maxima (α, β, δ bands) appear around 570, 540, and 360 nm, respectively. As an example, the so far unpublished UV–Vis-spectrum of the UPO of *Cyclocybe* (*Agrocybe) parasitica* is shown in Figure 2, along with the electrophoretic characteristics of the corresponding protein.

After reduction of UPO-heme (Fe^3+^ → Fe^2+^) with sodium dithionite and flushing with carbon monoxide (CO), the Soret band shifts towards a longer wavelength (445–448 nm) but without reaching the prototypical 450 nm mark of eponymous monooxygenases (P450s, CYPs) [2,5,29,75,76].

The crystal structures of UPOs from *A. aegerita* (wild-type, recombinant, and mutant proteins), *M. rotula*, and, very recently, from recombinant *Hypoxylon* sp. EC38 have been solved, revealing a compact spherical shape dominated by α-helices and containing a heme-stabilizing magnesium (Mg^2+^) and a highly conserved PCP motif [3,40,61,62,77]. The latter perfectly exposes a cysteine flanked by two ‘chain-breaking’ prolines towards the heme iron. The heme access channels of UPOs are lined with hydrophobic amino acid residues (e.g., Phe or Leu/Ile/Val) and their molecular architecture is crucial for the substrate specificity of the respective enzyme [29,35,38].

**Figure 3:**
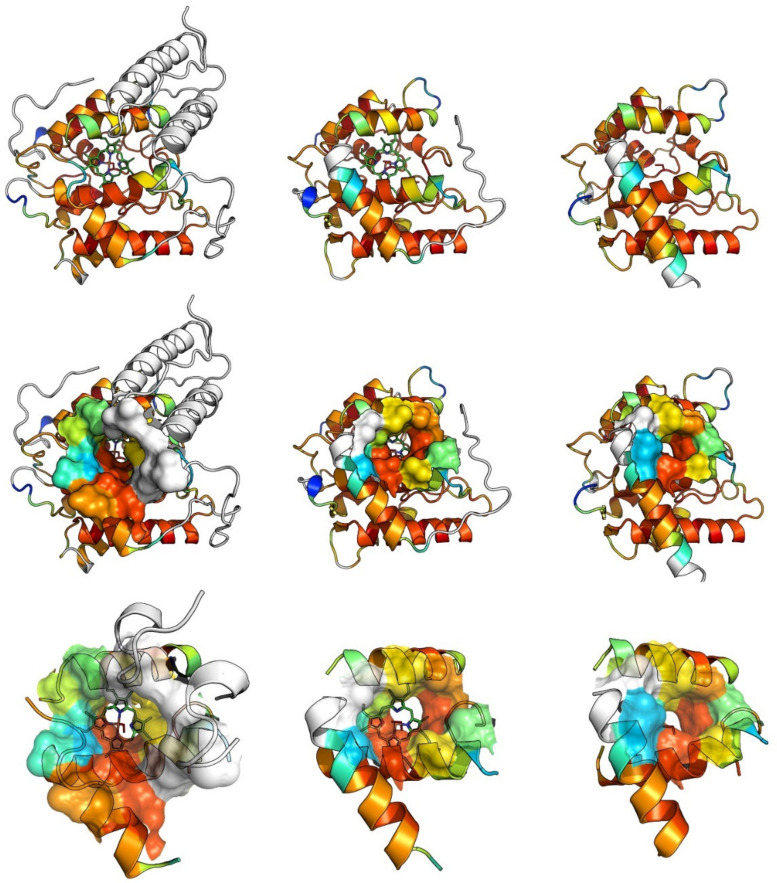
Models of three unspecific peroxygenases (UPOs). Left: *Cyclocybe* (*Agrocybe*) *aegerita*—*Aae*UPO, middle: *Marasmius rotula*—*Mro*UPO, right: *Chaetomium globosum*—*Cgl*UPO. Upper row: ribbon representation, middle row: mixed surface and ribbon representation, lower row: heme access channels. To predict the tertiary structure of *Cgl*UPO, the ColabFold [78] ‘AlphaFold2_mmseqs2’ notebook was used (accessed Nov 2021). Both amber relaxation/refinement and the usage of templates were selected and the input sequence was queried vs. the ‘UniRef’+’Environmental’ databases. The Alphafold2_mmseqs2 notebook is a variation of the AlphaFold2 prediction architecture [79]. PyMOL (PyMOL Molecular Graphics System, Version 2.5.2 Schrödinger, LLC) was used to visualize the resolved protein structures of *Aae*UPO (PDB#: 2YOR chain A), *Mr*oUPO (PDB#: 5FUK chain A), as well as the highest-ranked predicted structure of *Cgl*UPO. The PyMOL plugin PyMOD3.0 [80] was used to align the proteins with the built-in SALIGN module of the MODELLER [81] package. To find structurally conserved regions (SCRs) between the three proteins, the SCR_FIND algorithm [82] was used on the alignment of SALIGN (SC score limit = 3.0, sliding window size = 3, gap penalty = 100). SCR_FIND colors residues according to the rainbow colors, ranging from blue (low structural conservation) to red (higher structural conservation). Residues that were not found to be conserved among all proteins are shown in white.

Three examples of such structural models of UPOs can be found in Figure 3.

Meanwhile, 3D modeling based on primary sequences and known crystal structures has reached such quality that it can be used to obtain reliable protein structures [83,84]. 

Most UPOs are mono[per]oxygenases acting outside the fungal cells and transferring a peroxide-borne oxygen atom (H-O-O-R) to diverse organic substrates. The target substrates are subject to hydroxylation, epoxidation, and heteroatom oxygenation, and, in turn, to spontaneous dealkylation, deacylation, or (re-)aromatization [25].

**Figure 4:**
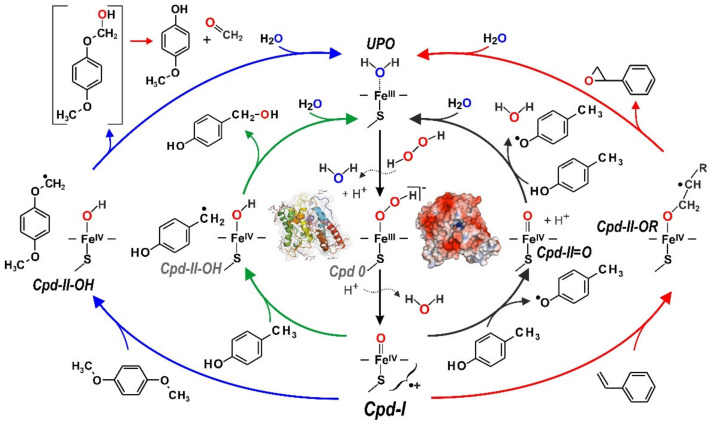
Catalytic cycles of an unspecific peroxygenase (UPO). Using three different substrates (*p*-cresol, *p*-hydroxyanisole, styrene), the scheme shows three possible oxygenations and a one-electron oxidation, which can be catalyzed by a single UPO, e.g., by the model enzyme from *Cyclocybe* (*Agrocybe*) *aegerita* (*Aae*UPO). Left: benzylic hydroxylation of *p*-cresol to 4-hydroxybenzyl alcohol (inner green arrows), *O*-dealkylation of 1,4-dimethoxybenzene to *p*-hydroxyanisole (outer blue arrows) via an unstable hemiacetal intermediate (in square brackets); right: epoxidation of styrene to styrene oxide (outer red arrows) and oxidation of *p*-cresole to two corresponding phenoxy radicals (inner black arrows). All reactions proceed via a short-lived compound 0 (Cpd-0) state and reactive compound I (Cpd-I) as the key intermediate but differ regarding the compound II states (Cpd-II). While hydroxylation and *O*-dealkylation proceed via protonated compound II (Cpd-OH), epoxidation requires the transitional formation of a compound II that binds the substrate as C-radical (Cpd-II-OR^•^) and the second phenolic substrate molecule is oxidized by peroxidase-typical, deprotonated compound II (Cpd-II = O) (modified according to [8,11,28,29,87]).

In addition, UPOs catalyze one-electron oxidations, i.e., the abstraction of single electrons (mostly together with protons) from susceptible functionalities such as phenolic hydroxyl groups, analogous to conventional peroxidases (Figure 4). Often, the product spectra of UPOs, e.g., regarding the oxidation of organopollutants and drugs, resemble those of cytochrome P450 monooxygenases (P450s, CYPs) that act, among others, as intracellular detoxification enzymes in the liver of vertebrates [7,85,86].

Figure 4 illustrates the mechanisms of UPO-catalyzed oxygenation and oxidation reactions by the exemplary substrates *p*-cresol (4-methylphenol), 1,4-dimethoxybenzene (*p*-methoxyanisole), and styrene (vinylbenzene). The former undergoes both benzylic hydroxylation and oxidation to the corresponding phenoxy radical [10,88], the aromatic diether is *O*-dealkylated (via an unstable hemiacetal intermediate) [89], and styrene’s side chain is subject to epoxidation [90]. The reactive UPO key species is, in each case, the so-called compound I (Cpd-I), a high-valent oxoferryl cation π-radical complex of heme, [heme]^•+^-Fe^4+^ = O, that is formed after the binding and heterolytic cleavage of H_2_O_2_ [11,91]. UPO Cpd-I is an extremely strong oxidant that attacks C-H bonds (sp^3^), C=C bonds (sp^2^, π-systems and aromatics), S-/N-heteroatoms, and phenolic OH-groups via radical formation (C^•^, S^•^, N^•^, O^•^). Subsequently, rebound mechanisms lead to the transfer of oxygen (peroxygenation of a C/S/N atom or epoxide formation) or to the formation of a second O^•^ radical (phenol oxidation) [29]. The reactions taking place thus correspond to a combination of the so-called ‘peroxide shunt’ of certain P450s and the catalytic cycle of typical heme peroxidases [92].

## 4. Selected Reactions Catalyzed by UPOs

**Figure 5:**
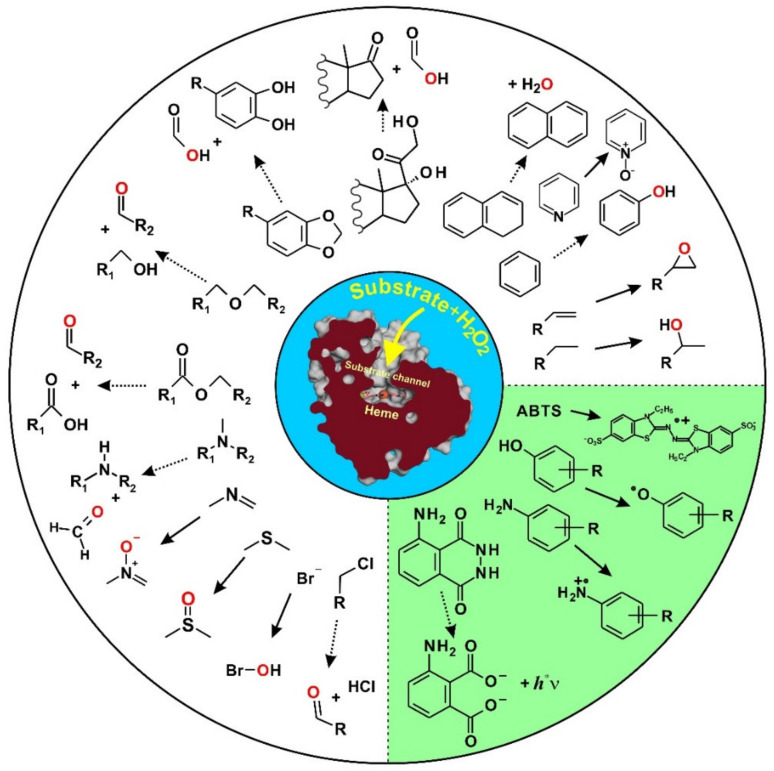
Reactions catalyzed by unspecific peroxygenases (UPOs). Reactions on the green background are one-electron oxidations resulting in unstable products (radicals). All other reactions are related to oxygen transfer from peroxide to diverse target substrates, resulting in hydroxylated, epoxidized, or cleaved products, modified according to [2,25,29].

UPOs catalyze diverse oxygen transfer reactions in the peroxygenase mode, including the hydroxylation of sp^3^-carbons (C-H) and epoxidation of sp^2^-carbons (C=C), some of which may undergo spontaneous further reactions, such as *O*- and *N*-dealkylation, epoxide opening, deacylation, or aromatization. Heteroatoms in organic molecules, such as sulfur (C-S) and nitrogen (C-N), are the subjects of oxygen transfer as well, and inorganic halides (X^−^) can be oxidized into hypohalites (OX^−^), which in turn halogenate other molecules (haloperoxidase mode). In the ‘classic’ peroxidase mode, UPOs oxidize prototypical substrates such as ABTS, phenolics, aromatic amines, and luminol by one-electron oxidations [28,29]. Figure 5 summarizes UPO-catalyzed reactions in a simplified scheme; an overview of the kinetic properties of UPOs, whose catalytic efficiencies (k_cat_/K_m_) for oxygenations range between 10^3^ and 10^6^ M^−1^ s^−1^, is given in Hofrichter et al. (2015) [2].

Only a small group of UPOs have been demonstrated to catalyze a range of oxygenations at different types of carbons, which is reflected by numerous original papers (>80) and review articles dealing with UPO-catalyzed conversions since their discovery in 2004; some relevant review articles covering, in addition to biochemical and synthetic, also biosensor-related findings can be found in [2,7,10,24,25,29,30,31,93,94,95,96,97,98,99,100,101].

**Figure 6:**
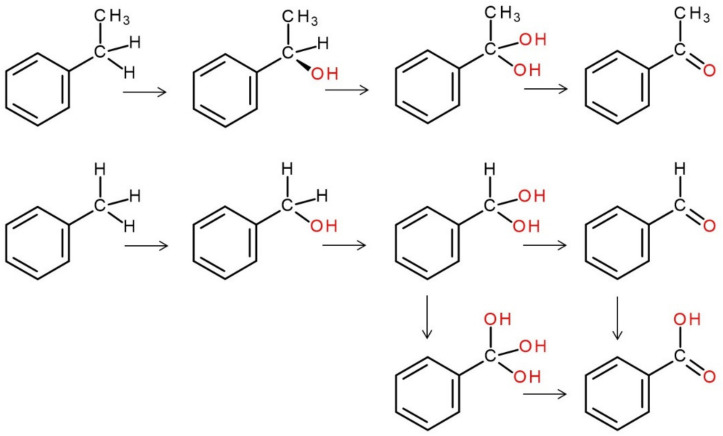
UPO-catalyzed, stepwise oxidation of benzylic carbon by the examples of ethylbenzene (above) and toluene (below), resulting in the formation of acetophenone and benzoic acid, respectively. The oxygen originating from peroxide (H_2_O_2_) is marked in red.

Typically, the UPO-mediated oxidation of C atoms (e.g., of an appropriate alkyl moiety such as benzylic CH_3_ or CH_2_-R) begins with the insertion of oxygen from hydrogen peroxide (H-O-O-H), resulting in the corresponding alcohols (CH_2_-OH, CH_2_-OH-R). These primary and secondary alcohols can be subject to further hydroxylation at the same carbons, which leads to the formation of the corresponding *gem*-diols [carbonyl hydrates CH-(OH)_2_], which are in equilibrium with aldehydes (CHO) and ketones (C=O), respectively. The aldehyde functionality in turn can be hydroxylated again to form the corresponding COOH group (carboxylic acid) (Figure 6). The first hydroxylation may proceed entantioselectively, as in the case of ethylbenzene and *Aae*UPO, affording preferably (*R*)-1-phenylethanol [90,102].

In the case of less reactive alkanes, most UPOs favor the hydroxylation at secondary and tertiary (as far as sterically possible, e.g., in isobutane) carbons over that of terminal carbons, which is due to different C-H bond dissociation energies [11,103]. Thus, linear alkanes (propane to *n*-hexadecane) are preferably oxidized to form alcohols with the hydroxyl group in the 2- or 3-position. Notably, *Mro*UPO was found to oxygenate the unfavored terminal carbon of long-chain *n*-alkanes (C_12_-C_14_) when ten-times-higher concentrations of H_2_O_2_ were applied. This led to mixtures of alcohols, ketones, and acids, including biotechnologically relevant dicarboxylic acids [62]. *Aae*UPO was also found to hydroxylate various cycloalkanes from cyclopentane to cyclooctane, yielding mainly the corresponding cycloalkanols and smaller amounts of cyclic ketones [103,104].

**Figure 7:**
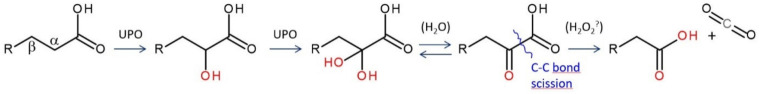
Shortening of fatty acids by stepwise oxidation of the C_α_ carbon, resulting in the formation of an α-keto derivative that spontaneously shortens via decarboxylation. The question mark at H_2_O_2_ indicates that it is unclear to what extent it contributes to final C-C scission.

In fatty acids, representing amphiphilic alkyls, the rather ‘unpolar’ positions ω-1 and ω-2 are favored by *Aae*UPO [12]. In contrast, Olmedo et al. (2017) [62] demonstrated that *Mro*UPO catalyzes the attack of the C_α_ directly adjacent to the polar carboxylic group, which results in the shortening of fatty acids. This interesting reaction sequence (Figure 7) requires the incipient oxygenation of the fatty acids’ C_α_ position, which, after a second hydroxylation, forms a *gem*-diol, being in equilibrium with the corresponding α-keto fatty acid. Finally, a spontaneous, probably peroxide-forced oxidation yields the C_1_-shortened fatty acid (a decarboxylation that results in CO_2_ and a new COOH group). In analogy to the well-known β-oxidation, this pathway could be referred to as α-oxidation of fatty acids. Similar consecutive peroxygenations and spontaneous oxidations have been reported for the deacylation (side chain removal) of corticosteroids [35].

**Figure 8:**
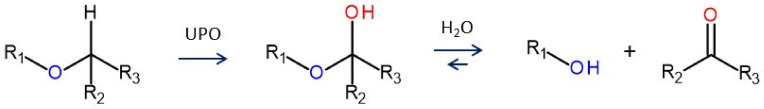
Ether scission (*O*-dealkylation) via a hemiacetal intermediate, resulting in the formation of an alcohol and a carbonyl (R_2_ and R_3_ = organic rests → ketone, R_2_ or R_3_ = H → aldehyde).

The scission of ethers (*O*-dealkylation) is another UPO-catalyzed reaction sequence that starts with carbon oxygenation, namely of the ether bond’s adjacent methylene (R-O-CH_2_-R’) or methyl (R-O-CH_3_) groups. We first demonstrated this reaction type using *Aae*UPO and a representative selection of substrates, including linear, branched, and cyclic ethers (e.g., diethyl ether, diisopropyl ether, tetrahydrofuran, 1,4-dimethoxybenzene) [89]. Upon hydroxylation, a hemiacetal is formed (e.g., R_1_-O-CH_2_-OH-R_2_) that spontaneously cleaves (actually, the hemiacetal is in equilibrium with the corresponding alcohol and aldehyde, similar to carbonyl hydrates (*gem*-diols) with water and free carbonyls) into an alcohol (R_1_-OH) and an aldehyde (O = CH_2_-R_2_), in which the alcohol bears the oxygen of the original ether and the carbonyl that of UPO-catalyzed oxygenation (Figure 8).

The oxidative fission of an ester, as a special case of *O*-dealkylation, was observed only for one UPO from the ink-cap *Coprinellus radians* (*Cra*UPO), which cleaved the antiviral drug osaltemivir in an H_2_O_2_-dependent manner [85]. Analogously to *O*-dealkylations, UPOs catalyze also *N*-dealkylations, with hemiaminals as putative intermediates. This was demonstrated for some alkylated anilines and various human drugs, including bulky pharmaceuticals such as SAR548304 (volixibat) [85,88,105].

**Figure 9:**
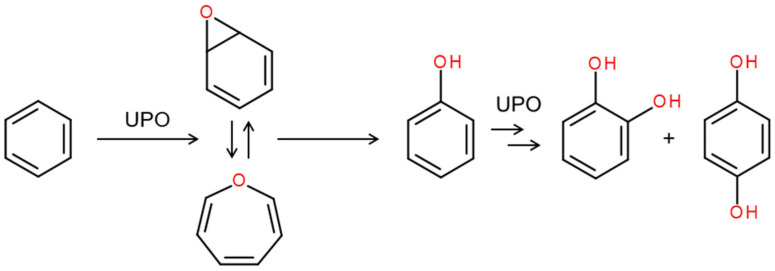
Epoxidation of benzene and subsequent spontaneous isomerization of benzene oxide under re-aromatization to form phenol. The second attack gives catechol and hydroquinone (modified according to [106]). The reaction sequence was confirmed using ^18^O-enriched hydrogen peroxide (H_2_^18^O_2_) as a co-substrate. Note, both benzene oxide and oxepine were detectable by HPLC.

Benzene, as the prototypical arene molecule, may serve as an example for the attack of the relatively stable aromatic ring. *Aae*UPO was found to oxygenate this non-activated sp^2^ system substantially [106]. Phenol (hydroxybenzene), catechol (1,2-dihydroxybenzene), and hydroquinone (1,4-dihydroxybenzene) were the major products of this reaction (additionally, traces of trihydroxybenzenes can be formed) (Figure 9). Long UPOs such as *Aae*UPO (and, to some extent, *Cra*UPO) may even favor this reaction over side-chain oxidation (as in the case of toluene, stelbenoids, and several pharmaceuticals), while short UPOs (e.g., *Mro*UPO) hardly oxidize the benzene ring or related structures [107]. It can be assumed that all ‘aromatic hydroxylations’ proceed via an initial epoxidation to the corresponding arene oxide (epoxyarene), though this was explicitly demonstrated only for benzene and naphthalene as substrates and *Aae*UPO as an enzyme [106,108]. In the case of benzene, the epoxyarene is in equilibrium with the corresponding oxepine and, due to the force of aromatization, the spontaneous isomerization of the epoxide follows epoxidation, giving rise to the corresponding phenols. This type of oxyfunctionalization was also shown for various substituted monoaromatic compounds and polycyclic aromatic hydrocarbons up to benzo(*a*)pyrene [49,85,86,109].

**Figure 10:**
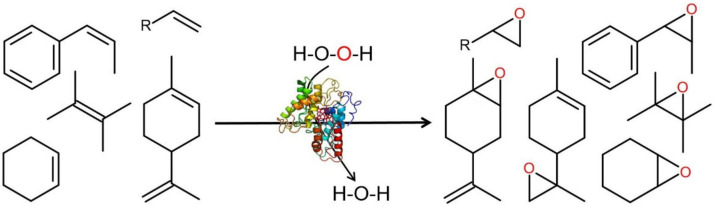
Epoxidation of aliphatic and cycloaliphatic alkenes (attack on sp^2^ carbons). Left side: (*cis*-methylstyrene, 1-alkene (R = C_1_-C_6_), cyclohexene, limonene; right side: 1,2-alkene oxide (2-alkyloxirane), *cis*-methylstyrene oxide (2-methyl-3-phenyloxiranre), limonene 6,7-oxide (4-isopropenyl-1-methoxy-1-methyl-cyclohexene), limonene 2,9-oxide [4-(2-methoxy-1-methyl-ethyl)-1-methyl-cyclohexene], (2,2,3,3-tetramethyloxirane), cyclohexene oxide (7-oxabicyclo[4.1.0]heptane).

The oxygenation of aliphatic and alicyclic alkenes (e.g., propene, *trans*-butene, cyclohexene) follows a similar scheme, resulting in respective epoxides (oxiranes) (Figure 10). However, due to a lacking driving force, the rearrangement of these epoxides is not promoted and they remain relatively stable [110]. Epoxidation of aliphatic sp^2^ carbons is catalyzed by all available UPOs (including CPO) and functions with a broad range of substrates, from simple and branched alkenes, via unsaturated fatty acids, to cyclic alkenes including complex terpenes and steroids [38,111,112,113,114,115].

**Figure 11:**
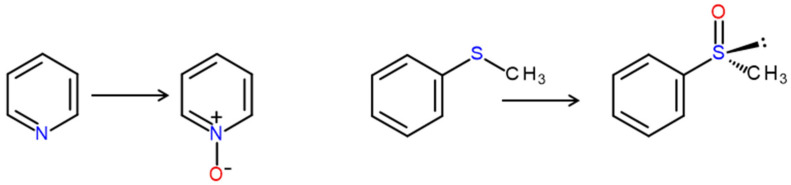
Oxygenation of pyridine’s heterocyclic nitrogen (left reaction) and of thioanisole (right), resulting in the formation of pyridine *N*-oxide and (*R*)-phenyl methyl sulfoxide [(*R*)-methylsulfinylbenzene], respectively.

In addition to the oxygenation of carbon atoms (C), UPOs were shown to oxygenate organic heteroatoms such as sulfur (S) and nitrogen (N) (Figure 11). In the latter case, they favor *N*-dealkylation over *N*-oxidation, if the nitrogen is substituted with alkyls (e.g., methyl group). In contrast, aromatic nitrogen as found in pyridine is subject to *N*-oxidation [116]. Pyridine and its derivatives are relatively electron-deficient and thus inactivated regarding electrophilic attack. Hence, it is plausible that the yields of pyridine *N*-oxides are rather poor in UPO conversions. The formation of phenolic products (i.e., pyridinoles = hydroxypyridines) has never been observed. It is assumed that the heterocyclic nitrogen tends to coordinate towards the heme iron so that its oxidation is favored over that of carbons. There is also an indication that *Aae*UPO can attack the amino group in anilines via *N*-oxidation, resulting in the corresponding hydroxylamino derivatives; however, the major products of aniline oxidation are aminophenols [117,118].

*Aae*UPO catalyzes also sulfoxidations, which were thoroughly studied by the example of thioanisole and related aryl-alkyl sulfides that were also oxidized in deep eutectic solvents [119,120]. In all cases, the absolute configuration of the resulting sulfoxides was (*R*) and the enantiomeric excess reached up to >99% in dependence on the alkyl substituent and the reaction conditions. Besides aryl-alkyl model sulfides, sulfoxidations were also reported for complex molecule such as the pharmaceutical omeprazole and the crude oil constituent dibenzothiophene [85,121].

UPOs (e.g., *Aae*UPO, *Cra*UPO, r*Cci*UPO) chlorinate organic compounds only poorly (in contrast to their ‘sister enzyme’ chloroperoxidase), but most of them show brominating activities for phenolic substrates [36,41,122]. Notably, *Mro*UPO was found to neither chlorinate nor brominate phenolic substrates [34]. The most plausible reaction mechanism includes the formation of reactive hypohalites (OX^−^) as primary oxygenation products that in turn attack preferably sp^2^-hybridized carbons [10,123].

(i) X^−^ + H_2_O_2_ → OX^−^ + H_2_O (ii) OX^−^ + R-H → HX + R-X R = phenyl, X = Br, (Cl)

There is high variation in the halogenating activities of UPOs, but the molecular basics that guide these reactions, particularly regarding their selectivity, are not fully understood.

More than a decade of intensive studies on the reactions of UPOs, of course, also revealed several limitations in the general performance of these enzymes. The most important constraints are: (a) steric hindrance and too large substrates, (b) the absence of abstractable hydrogen atoms at the carbon(s) to be attacked, and (c) too high C-H bond dissociation energies (BDEs). Steric constraints that limit the substrate spectrum of UPOs to smaller substrates are not surprising, since the actual oxygenation requires the direct contact of at least part of the substrate with the ferryl oxygen (Fe^4+^ = O) of the activated heme, UPO Cpd-I, which requires their passage through the heme access channel. Thus, larger and compact molecules such as perylene (5-ring PAH, C_20_H_12_) and cyclodecane (C_10_H_20_) were oxidized neither by *Aae*UPO nor by *Cra*UPO [109,112]. The same applies to polymeric lignin, although a non-phenolic lignin dimer was oxidized by *Aae*UPO [117,124]. Whether UPOs with rather wide and shallow heme access channels exist (or can be engineered), which allow heme to contact bulky polymeric substrates such as lignin (analogous to the interaction of the copper centers of lytic polysaccharide monooxygenases (LPMOs) with crystalline cellulose) [125], will be an intriguing question for the future. Furthermore, biphenyl ethers cannot undergo ether scission because of the absence of hydrogens at the ether oxygen’s adjacent carbons. Moreover, 2,2,3,3-tetramethylbutane is a further example of a molecule that cannot be attacked by UPOs due to the lack of abstractable hydrogens. The molecule contains six terminal carbons that cannot be oxidized because of the high C-H BDEs, and two carbons (at positions 2 and 3) that do not bear any hydrogen [112]. Eventually, methane, with its exceptionally high C-H BDE (105 kcal mol^−1^), was not converted by *Aae*UPO [112], which is in accordance with the BDE vs. log k plot for *Aae*UPO Cpd-I [11].

Despite these obvious limitations, we anticipate that new UPO-catalyzed reactions will be found in the coming years, particularly if the large-scale production of diverse recombinant UPOs succeeds. One example from our current research is the cleavage of activated aromatic rings as found in catechol, which mimics a dioxygenase-type reaction.

## 5. Occurrence in the Fungal Kingdom and Phylogeny

We have meanwhile found several thousand putative UPO sequences in databases and fungal genomes, more than 4000 alone in NCBI, corresponding to more than 8000 in JGI Mycocosm (i.e., out of 2156 genome-sequenced fungi, UPOs are present in 1656 fungi; blast search from November 2021) and several hundred from our own unpublished genome sequencing approaches [72]. This suggests the wide distribution of these enzymes throughout the fungal kingdom, which includes all phyla of true fungi (*Eumycota*) as well as a number of fungus-like *Stramenopiles* [2,29]. Within the most basal sister group of all other fungi, the *Cryptomycota*, one UPO gene was identified in *Rozella allomycis*, an obligate endoparasite in other primordial fungi and fungus-like protists [126]. Notably, other basal fungal groups (*Microsporidia*, *Neocallimastigomycota*, *Blastocladiomycota*, *Kickxellomycotina*) lack UPO genes (according to the genomes available so far); the same is true for the sister group of fungi, the *Holozoa*, which includes animals (*Metazoa*) and their closest relatives (*Choanoflagellata*, *Ichthyosporea*). Representatives of the *Chytridiomycota* (‘flagellate fungi’), on the other hand, possess up to seven UPO genes. Thus, their enzymes may have been the starting point for the evolutionary development of the UPO multigene family, which eventually led to the possession of 80 or more UPO genes in an individual species of ‘higher fungi’ [29]. Apart from the *Mortierellomycotina*, sequenced representatives of the other groups of the polyphyletic ‘*Zygomycota’* and *Glomeromycota* possess one to seven UPO genes.

Within the species-rich subkingdom of ‘higher fungi’ (*Dikarya*, i.e., *Ascomycota* and *Basidiomycota*), UPO genes are particularly abundant. They reach their greatest number within the *Ascomycota* in the citrus pathogen *Zasmidium citri-griseum* (up to 33 genes) and in the halotolerant black yeast *Hortaea werneckii* (up to 25 genes) [29]. Within the *Basidiomycota*, species from different eco-physiological fungal groups have multiple UPO genes, especially common among decomposers of soil leaf litter, compost, and dung. For example, the ‘Cannon ball fungus’ (*Sphaerobolus stellatus*), which grows on woody debris and manure, possesses no fewer than 78 UPO genes. The record holder, for the time being (12/2021), is *Mycena rebaudengoi* (leaf litter and twig decomposer), with no less than 83 UPO genes. In our own, yet unpublished, studies, we have found over 60 UPO genes in the ‘Pinwheel mushroom’ (*Marasmius rotula*) and 48 genes in the newly described Kenyan species *Candolleomyces* (*Psathyrella*) *aberdarensis* [2,29]; both fungi preferentially colonize small woody debris as well. The most important commercial edible fungus, the ‘White button mushroom’ (*Agaricus bisporus*), which is routinely grown on nitrogen-rich compost materials, is also rich in UPOs (24 genes) [127,128]. Putative UPO genes outside of the true fungi (*Eumycota*) have only been found in various *Peronosporomycetes* (formerly ‘*Oomycota’*), two diatoms, and in one slime-mold-like organism (*Planoprotostelium fungivorum*). In the latter two cases, the assignment is uncertain and must be confirmed by additional genomes of related species in the *Bacillariophyta* (SAR, *Stramenopiles*-*Alveolata*-*Rhizaria* supergroup) and *Amoebozoa* (sister supergroup of *Eumycota* and *Metazoa*/animals). *Peronosporomycetes* are fungus-like *Stramenopiles* that are phylogenetically closer to colored algae (e.g., *Phaeophyta*) than to true fungi. Corresponding UPO genes have been detected, among others, in the pathogens *Aphanomyces astaci* (crayfish plague) and *Phytophthora infestans* (potato blight, eleven UPO genes). There is evidence that these pseudo-fungi, also known as ‘water molds’ (having up to 24 UPO genes as in a *Neophytophtora* sp.), obtained them multiple times via horizontal gene transfer from pathogenic *Ascomycota* [129,130]. As another phylogenetic trend, we have found that certain yeasts and yeast-like fungi with reduced genomes within the *Ascomycota* and *Basidiomycota* lack UPO genes (*Saccharomycotina*, *Schizosaccharomycetes*, *Malasseziamycetes*).

**Figure 12:**
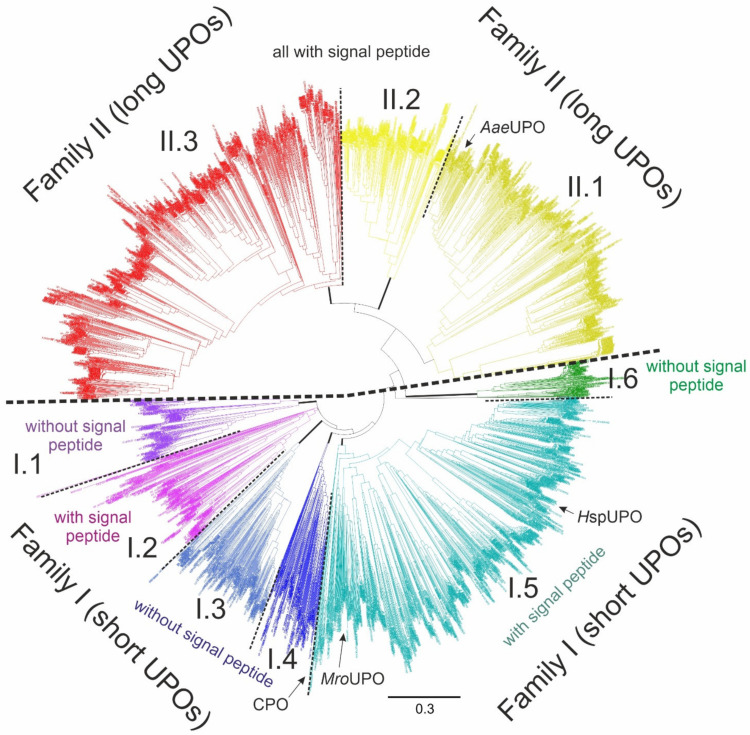
Neighbor-joining phylogenetic tree of 3728 UPO/HTP sequences using genetic distances calculated with Geneious Prime 2021. Classification into short and long UPOs (families I and II, respectively) as well as into clearly distinguishable clades (subfamilies I.1-I.6 and II.1-II.3) is illustrated by different colors and black dashed lines. UPO sequences with resolved crystal structure are marked with arrows (*Aae*UPO—*Cyclocybe aegerita* PDB: 2YOR, *H*spUPO—*Hypoxylon* sp. EC38 PDB: 7O2G, *Mro*UPO—*Marasmius rotula* PDB: 5FUK, CPO—*Leptoxyphium* (*Caldariomyces*) *fumago* PDB: 1CPO). UPO genes of indicated thick branches are composed identically using a maximum-likelihood approach (FastTree 2.1.11 [131], 20 rate categories). Clades of putatively secreted UPOs carrying signal peptides and probably intracellular UPOs without such sequences are labeled with appropriate text. The nine distinguishable clades comprise fungal UPO genes of different taxonomic affiliation (on phyla and subphyla levels): clade I.1—*Ascomycota*, *Cryptomycota*; clade I.2—*Ascomycota*, *Basidiomycota*, *Chytridiomycota*, *Mucoromycotina*, *Oomycetes*, *Zoopagomycota*; clade I.3—*Ascomycota*, *Basidiomycota*; clade I.4—*Basidiomycota*; clade I.5—*Ascomycota*, *Basidiomycota*; clade I.6—*Ascomycota*; clade II.1—*Ascomycota*, *Basidiomycota*; clade II.2—*Ascomycota*, *Basidiomycota*; clade II.3—*Ascomycota*, *Basidiomycota*.

Phylogenetically, UPO sequences can be divided into two protein families. Family I—the ‘short’ UPO sequences—includes representatives of all the above-mentioned UPO-containing phylogenetic groups of fungi (including *Peronosporomycetes*). Family II houses the ‘long’ UPOs, whose occurrence is restricted to the *Dikarya*, i.e., *Ascomycota* and *Basidiomycota* (Figure 12). Some distinct basal subgroups in family I contain UPO sequences without recognizable signal peptides or subcellular localization signals, so it seems plausible that the UPO archetype had been an intracellular enzyme. In total, this concerns approximately 30% of the analyzed UPO sequences (Figure 12). Whether these enzymes function freely in the cytosol, act in specific hyphal compartments, or are secreted by alternative pathways (e.g., exocytose vesicles) is unclear. The current UPO phylogeny using a distance and maximum-likelihood approach displays a finer branching than previous attempts [2,29]. The ‘short’ UPOs seem to diversify into several subfamilies, some without a signal peptide, hereby including all basal fungal taxa (Figure 12).

Since UPOs are found exclusively in the fungal kingdom [132], including the basal *Cryptomycota*, and no evidence for their presence in the *Holozoa* has yet been found, it can be postulated that they originated more than 600 million years ago (late Proterozoic), after the splitting of *Opisthokonta* into their major groups (*Eumycota* and *Holozoa*/*Metazoa*). It seems plausible that the drastic conditions of the second evolving O_2_ atmosphere during this time (around 700–550 million years ago) played an evolutionary role in this process, similar to the case of P450 enzymes. Whether UPOs and P450s have a common origin is still unclear, but, if so, it might date back to more than a billion years ago (before the separation of the major eukaryote lineages) [133]. Although both enzyme types share structural and catalytic similarities (cysteine as proximal heme ligand, reactive heme intermediates, i.e., compounds 0, I, II), there is almost no homology (<5%) at the sequence level and hardly any similarity in the tertiary protein structure (arrangement of α-helices) [134], which rather points to the convergent evolutionary development of UPOs and P450s under similar environmental pressure [8,135]. In fungal organisms where both enzyme types occur, it may plausible that there is a division of labor between P450 enzymes and peroxygenases, in which one works inside hyphae and the other outside.

## 6. Conclusions and Outlook 

Despite their high relevance for synthetic applications in (bio)organic chemistry, only a few UPOs are available so far and none of them in economically relevant amounts. Furthermore, almost nothing is known about their natural functions, which is not merely an academic question but also important regarding biotechnological attempts and eco-physiological considerations. Taking into account their wide distribution in the fungal kingdom, their high number in the genomes of certain fungi, and their promiscuity in terms of catalyzed reactions, different functions are conceivable. The first would certainly be detoxification reactions, as UPOs attack structures commonly found in plant and microbial secondary metabolites and pharmaceuticals, as well as environmental pollutants [7,8,136]. Bearing in mind the high diversity of UPO genes, involvement in pathogenicity events or in lignin and humus transformation should also not be excluded. For the latter, a molecular architecture would be required that allows the superficial targeting of aromatic polymer structures via heme access channels that are as broad and shallow as possible. In this context, it should be noted that, according to recent publications, the crystalline cellulose-cleaving LPMOs (lytic polysaccharide monooxygenases), which were discovered only a few years ago, are also surface-acting extracellular peroxygenases (albeit based on copper-dependent catalysis) [125,137,138].

Modern molecular techniques such as genome editing using CRISPR/Cas could help to answer the question regarding the function of UPOs. For this, UPO knock-out mutants of suitable model organisms would need to be generated and subsequently physiologically tested. Molecular and enzymatic field studies (e.g., in the context of the Biodiversity Exploratories, DFG SPP-1374) could also help to clarify the functions of UPOs—for example, in a manner similar to that achieved in the case of manganese peroxidases [139,140]. In order to accelerate the production of recombinant UPOs, cell-free expression with isolated ribosomes could be an innovative approach, in addition to the further development of all types of microbial and other expression systems (yeasts, molds, bacteria, plant and insect cells, etc.).
